# Standard treatment–refractory cytomegalovirus encephalitis unmasked by immune reconstitution inflammatory syndrome and successfully treated with virus‐specific hyperimmune globulin

**DOI:** 10.1002/cti2.1201

**Published:** 2020-11-17

**Authors:** Natalia Maximova, Annalisa Marcuzzi, Irene Del Rizzo, Davide Zanon, Alessandra Maestro, Egidio Barbi, Roberto Sala

**Affiliations:** ^1^ Institute for Maternal and Child Health – IRCCS Burlo Garofolo Trieste Italy; ^2^ University of Ferrara Ferrara Italy; ^3^ University of Trieste Trieste Italy; ^4^ University of Parma Parma Italy

**Keywords:** cerebrospinal fluid CMV antibodies, CMV‐hyperimmune globulin, cytomegalovirus (CMV)‐related encephalitis, IL‐6, immune reconstitution inflammatory syndrome (IRIS)

## Abstract

**Objectives:**

Cytomegalovirus (CMV)‐related encephalitis is a rare but potentially life‐threatening complication of CMV infection in immunocompromised patients. The high mortality rate is associated with deficient immune system reconstitution after hematopoietic stem cell transplant (HSCT) and poor bioavailability of antiviral drugs in cerebrospinal fluid (CSF). CMV‐related central nervous system (CNS) infection may occur with aspecific symptoms, without evidence of either blood viral load or magnetic resonance imaging (MRI) signs of encephalitis.

**Methods:**

Here, we describe a 10‐year‐old girl who underwent an allogeneic HSCT and subsequently developed CMV encephalitis. Because of the absence of CMV antigen in the blood, the diagnosis of encephalitis was proposed only after a delay, following the onset of immune reconstitution inflammatory syndrome (IRIS). Two months of combined dual antiviral therapy with ganciclovir and foscarnet proved ineffective against CMV and caused significant bone marrow and renal toxicity. To avoid further toxicity, the girl was given daily treatment with CMV‐hyperimmune globulins alone.

**Results:**

After three weeks, the CSF viral load dropped significantly and was undetectable within three more weeks. In the meantime, the renal impairment resolved, and there was a complete bone marrow recovery.

**Conclusion:**

We suggest that this patient succeeded in achieving CMV CSF clearance with high dose of CMV‐hyperimmune globulin, given alone, because of the ability of immunoglobulins to penetrate the blood–brain barrier (BBB).

## Graphical abstract

This case describes the high‐dose anti‐cytomegalovirus (CMV) hyperimmune globulin treatment of CMV‐induced encephalitis after allogeneic hematopoietic stem cell transplant. After two months of combined dual antiviral therapy with ganciclovir and foscarnet, the CSF viral load remained very high; meanwhile, the patient, a 10‐year‐old girl, developed severe bone marrow and renal toxicity. Complete CSF clearance was obtained with high doses of anti‐CMV hyperimmune globulins given alone. We suggest this treatment succeeded because of the ability of immunoglobulins to penetrate the blood–brain barrier.

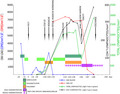

## Case report

**Figure 1:**
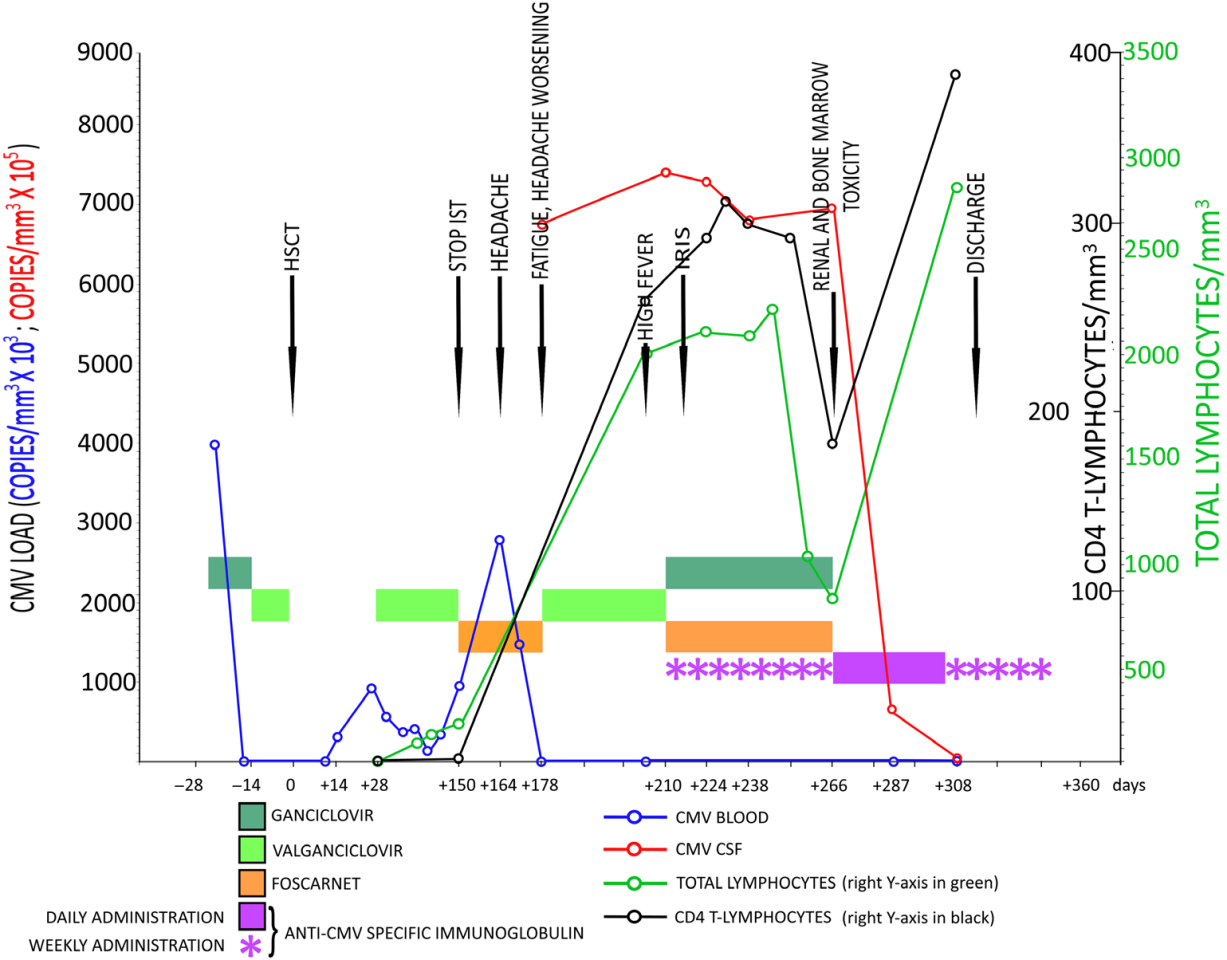
Lymphocyte count, cytomegalovirus (CMV) blood and cerebrospinal fluid (CSF) viral load, and the progression of symptoms and events over one year, in a 10‐year‐old girl who underwent allogeneic HSCT. Colour bars represent the timeline graph of antiviral treatments and their corresponding duration. After discontinuing the immune‐suppressive regimen, the patient first experienced headaches with high blood CMV load, despite therapy with valganciclovir and foscarnet. Her symptoms then worsened until overt immune reconstitution inflammatory syndrome (IRIS) occurred, with high CSF CMV load, despite CMV clearance from the blood, together with an increase in lymphocyte count and severe bone marrow and renal toxicity. After the initiation of anti‐CMV‐specific immune globulins, the CSF viral load dropped rapidly, and symptoms improved. HSCT, hematopoietic stem cell transplantation; IST, immunosuppressive therapy; IRIS, immune reconstitution inflammatory syndrome; CMV, cytomegalovirus; CSF, cerebrospinal fluid.

We present a 10‐year‐old girl who received allogeneic hematopoietic stem cell transplant (HSCT) for Philadelphia chromosome (Ph)‐positive acute lymphoblastic leukaemia (ALL) and subsequently developed cytomegalovirus (CMV) encephalitis, successfully treated with CMV‐hyperimmune globulin (Figure 1).

The girl was diagnosed with ALL one year prior, and as a result of the positivity for the Ph chromosome, she underwent HSCT in first complete remission. The virological screening performed during the pre‐transplant workup by quantitative polymerase chain reaction (PCR) showed a CMV viral load of 4.0 × 10^3^ copies mm^−3^. The patient received antiviral treatment with ganciclovir, obtaining a complete virus clearance in a few days. Immediately before the HSCT, ganciclovir was replaced with valganciclovir.

After total body irradiation (TBI)‐based myeloablative conditioning, the girl received 4.8 × 10^8^ kg^−1^ bone marrow total nuclear cells from the 10/10 HLA‐matched, CMV‐positive, unrelated donor. Graft‐versus‐host disease (GVHD) prophylaxis was performed with tacrolimus and mycophenolate mofetil. The patient received continuous antiviral prophylaxis with valganciclovir, except between days −1 and +20. Neutrophil and platelet engraftment were achieved at day +15 and day +22, respectively. The peripheral blood chimerism analysis at day +30 proved full donor chimerism, and the absence of BCR/ABL transcript in the bone marrow cells confirmed the complete molecular remission.

The patient could not start the maintenance treatment with tyrosine kinase inhibitors because of suboptimal graft function. She showed a marked lymphocyte depletion with a total lymphocyte count of 206 mm^–3^, CD3 4 mm^–3^, CD4 2 mm^–^^3^, CD8 1 mm^–^^3^ and CD19 4 mm^–^^3^ at day +150. For this reason, and because the loads of CMV, Epstein–Barr virus (EBV), human herpesvirus 6 (HHV‐6) and BKV remained low, the immunosuppressive regimen was discontinued.

At day +164, she developed a persistent headache and high blood CMV, EBV, HHV6 and BKV virus loads were detected. Valganciclovir was shifted to foscarnet. Magnetic resonance imaging (MRI) of the brain did not show central nervous system (CNS) involvement.

Despite the rapid viral blood clearance, the headache worsened, and severe fatigue developed. To rule out CNS disease relapse, at day +178, a lumbar puncture was performed. The cerebrospinal fluid (CSF) analysis revealed a lymphocytic pleocytosis (68 cells mm^−3^), slightly elevated protein level (49 mg dL^−1^) and normal glucose level (39 mg dL^−1^), without evidence of blast cells (CD19^+^CD58^+^CD10^++^) on flow cytometry. Tests for CSF toxoplasma, panfungal PCR and JC virus (JCV) were negative. The CSF specimen was stored at −80°C, pending some other diagnostic hypotheses, and the girl continued her outpatient antiviral treatment with valganciclovir.

At day +203, the patient presented with high fever and further worsening headache and fatigue. Lansky performance status decreased from 100% to 60%. A second MRI, serum C reactive protein (CRP) and all viral blood determinations were negative, while the total lymphocyte count increased to 2000 mm^–3^ with > 600 CD3 mm^−3^ and > 200 CD4 mm^−3^. At day +210, the girl was admitted because of fourth cranial nerve palsy with vertical diplopia and positive Bielschowsky test. A brain MRI showed multiple focal lesions with T1 hypointensity and diffusion hyperintensity without enhancement after contrasting medium administration. These findings were suggestive of an immune reconstitution inflammatory syndrome (IRIS). Quantitative PCR on CSF for CMV, EBV, all herpesviruses, JCV, toxoplasma and panfungal PCR was performed and showed a very high CMV load (7.3 × 10^5^ copies mm^−3^), whereas the CMV PCR on blood was negative. A CMV PCR was also performed on the frozen CSF sample, collected at day +178. The viral load in this sample was also very high (6.8 × 10^5^ copies mm^−3^). To detect why the clinical and radiological pictures were so different between the two episodes, despite the similar CSF viral loads in both specimens, CSF inflammatory cytokines concentrations were measured. IL‐6 value was very high (96.07 pg mL) only in the CSF sample collected during the onset of neurological symptoms, suggesting a robust immune restoration and a likely CMV‐induced IRIS.

The girl was started on a combined dual antiviral therapy with ganciclovir (10 mg kg^−1^ day^−1^), foscarnet (1800 mg kg^−1^ day^−1^) and weekly CMV‐hyperimmune globulin (Cytotect CP, Biotest, Germany) infusions (200 U kg^−1^ dose^−1^). All the neurological symptoms began to improve right away. Unfortunately, the CSF viral load did not drop significantly after two, four and eight weeks of dual therapy. The possibility of a drug‐resistant CMV strain was ruled out by the genotypic resistance test, which was aimed at detecting specific mutations in UL97 and UL54 genes.

At day +266, after two months of dual antiviral therapy, the girl developed severe renal impairment, neutropenia and thrombocytopenia. The CSF viral load was still very high, despite the complete clinical and radiological recovery. To avoid further renal and bone marrow toxicity, the dual antiviral therapy was suspended and Cytotect CP treatment alone was continued, at an increased frequency of 200 U kg^−1^ dose^−1^ for five consecutive days every week. At day +287, the CSF viral load dropped to 900 copies mm^−3^ and was undetectable at day +308. In the meantime, the renal impairment resolved, and there was a complete bone marrow recovery. On discharge, the girl was in excellent clinical condition, and she continued the CMV‐hyperimmune globulin prophylaxis once a week for two months as an outpatient. Five years out of HSCT, the girl remains in perfect health and has not experienced any further CMV reactivations. All data regarding blood and CSF viral loads are displayed in Table 1.

**Table 1:** Timing from HSCT of detection and quantification of Herpes Family virus and JCV in plasma and CSF samples

Time of Sampling^a^	CMV		HHV‐6		HSV1‐2		EBV		VZV		JCV
	plasma	CSF	plasma	CSF	plasma	CSF	plasma	CSF	plasma	CSF	CSF
− 21	4000	NP	NEG	NP	NEG	NP	NEG	NP	NEG	NP	NP
− 14	NEG	NP	NP	NP	NP	NP	NP	NP	NP	NP	NP
+ 14	340	NP	NEG	NP	NEG	NP	NEG	NP	NP	NP	NP
+ 150	1010	NP	560	NP	NEG	NP	920	NP	NEG	NP	NP
+ 164	2980	NP	2210	NP	NEG	NP	5730	NP	NEG	NP	NP
+ 178	NEG	680000	1670	NEG	NEG	NEG	3220	NEG	NEG	NEG	NEG
+ 210	NEG	730000	NEG	NEG	NEG	NEG	NEG	NEG	NEG	NEG	NEG
+ 224	NEG	710000	NEG	NEG	NEG	NEG	NEG	NEG	NEG	NEG	NEG
+ 238	NEG	683000	NEG	NEG	NEG	NEG	NEG	NEG	NEG	NEG	NEG
+ 266	NEG	694000	NEG	NEG	NEG	NEG	NEG	NEG	NEG	NEG	NEG
+ 287	NEG	900	NEG	NEG	NEG	NEG	NEG	NEG	NEG	NEG	NEG
+ 308	NEG	NEG	NEG	NEG	NEG	NEG	NEG	NEG	NEG	NEG	NEG

CMV, cytomegalovirus; CSF, cerebrospinal fluid; EBV, Epstein–Barr virus; HHV‐6, human herpesvirus 6; HSCT, hematopoietic stem cell transplantation; HSV, herpes simplex virus; JCV, JC virus; NEG, negative; NP, not performed; VZV, Varicella zoster virus.

^a^ Refers to days from HSCT.

**Figure 2:**
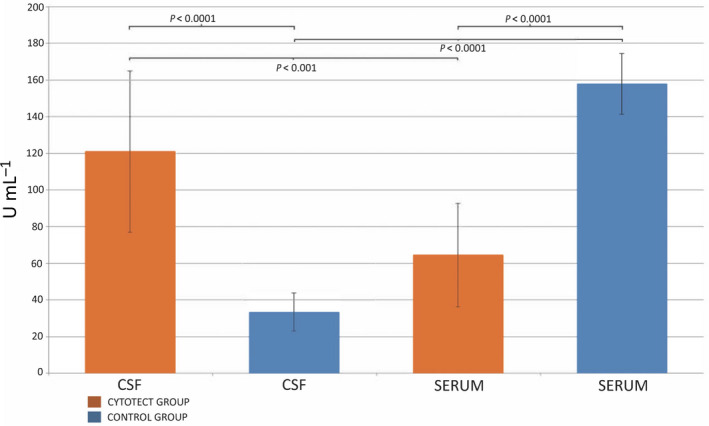
Serum and cerebrospinal fluid (CSF) cytomegalovirus (CMV)‐IgG antibodies (Ab) titre evaluation in CMV‐IgG‐positive patients undergoing the Cytotect CP treatment (Cytotect group, *n* = 15) and those not so treated (Control group, *n* = 15). In the Cytotect group, the CSF CMV Ab levels were significantly higher than serum Ab levels (*P* < 0.001) and CSF Ab levels of the Control group (*P* < 0.0001). CMV serum and CSF Ab evaluations were performed in triplicates, and their results are shown as mean ± SD. Statistical analysis was performed using the Mann–Whitney *U*‐test.

During the Cytotect CP treatment, we measured the CMV‐IgG antibody levels in the blood and CSF of our patient. The CNS antibody levels were significantly higher than those in blood in all the tests. For comparison, we conducted a retrospective analysis based on plasma and CSF samples stored in our biobank. From our records, we identified 30 CMV‐IgG‐positive paediatric patients affected by haematologic malignancies who had undergone standard treatment protocols and for whom both plasma and CSF samples were stored. None of these patients had CMV‐related CNS involvement. All samples were obtained 3–5 months post‐transplant. A minimum of three peripheral blood samples were collected for each patient. Except for the patient featured in this paper, there was only a single CSF sample for each of the patients. We divided selected patients into two comparable groups of 15 each. The patients in the first group had received prophylactic treatment with Cytotect CP (Cytotect group), while those in the second group had not (Control group). We performed serum and CSF CMV‐IgG antibodies titre evaluation on the frozen samples. Comparing the titres of CMV‐IgG antibody in blood and CSF, we found a statistically significant correlation in antibody concentration within each group, with an inverse correlation in the Cytotect group (*P* < 0.001 for the Cytotect group, *P* < 0.0001 for Control group). Comparing the titres of CMV‐IgG antibody in blood and CSF across both groups, a positive correlation was found in blood antibody concentration, while CSF antibody concentration showed an inverse correlation (*P* < 0.0001 for both). Comparing the CMV‐IgG antibodies blood/CSF ratio, we found statistically significant differences in antibody concentrations between the two groups, with inverse correlation in the Cytotect group (*P* < 0.001 for Cytotect group, *P* < 0.0001 for Control group). Similarly, comparing blood and CSF CMV‐IgG titres in both groups, the positive correlation was found in blood antibodies concentration, while CSF antibodies titre showed an inverse correlation (*P* < 0.0001 for both). The results are shown in Figure 2.

## Differential diagnosis

Early neurological complications of HCST, those that appear from the start of conditioning through the first month after transplant, may be a consequence of conditional regimen toxicity, CNS infections and haemorrhages during prolonged aplasia, immunosuppressant‐related neurotoxicity or proinflammatory state because of the release of cytokines.^1^, ^2^, ^3^, ^4^, ^5^ Late complications, occurring six months or more after HSCT, are more likely because of chronic GVHD, incomplete or aberrant regeneration of the immune system, secondary graft failure or recurrence of the primary disease.^6^ Complications occurring, as in our case, from two to six months from HSCT could have been because of some or all these conditions.

In this patient, a conditioning‐related complication was unlikely because of the lack of specific MRI features such as attenuation of the white matter, ventricular dilatation and intracerebral calcifications.^7^ Immunosuppressant‐related toxicity could be excluded because the onset of the symptoms began after the immunosuppressant treatment was discontinued. Cerebral toxoplasmosis and fungal brain abscess were ruled out because of negative microbiological and radiological assessments. Leukaemia relapse was excluded by flow cytometry analysis, while a CNS‐GVHD was highly unlikely because no other organs were involved.^8^, ^9^, ^10^

Immune reconstitution inflammatory syndrome is a clinical entity related to the immune system’s restoration, which follows immunosuppression after allogeneic HSCT, presenting as the unmasking of a clinically silent infection or as the worsening clinical manifestations of a pre‐existing infection.^11^ It should be ruled out in allogeneic HSCT recipients with neurological symptoms.^12^ In our case, the development of CNS‐IRIS allowed us to diagnose a previously evolved CMV encephalitis.

## Discussion

Cytomegalovirus‐related encephalitis is a rare but potentially life‐threatening complication of CMV infection in immunocompromised patients, including HIV‐infected patients, solid organ transplant recipients and HSCT recipients. With the improvement in highly active antiretroviral therapy (HAART), it occurs less frequently in HIV, whereas allogeneic HSCT recipients remain at significant risk.^13^

Neurologic complications after HSCT have a detrimental impact on survival.^14^ The high mortality rates are associated with deficient immune system reconstitution after HSCT and poor bioavailability of antiviral drugs in CSF.^15^ CMV‐specific T lymphocytes are critical in protecting against severe CMV disease after allogeneic HSCT. Still, most patients are severely lymphocyte‐depleted, since they receive either a T cell‐depleted stem cell graft or antithymocyte globulin during conditioning. Lastly, HSCT recipients frequently have a history of CMV viremia treated with pre‐emptive ganciclovir or foscarnet regimen, which may lead to the development of resistant strains.^16^

Cytomegalovirus‐related CNS infection may occur with aspecific symptoms, without evidence of either blood viral load or MRI signs of encephalitis. In the case of CMV infection, the difference between the viral load of blood compared with that of other tissues or body fluids is well recognized, particularly in gastrointestinal CMV disease.^17^ In addition, the absence of CMV in the blood of solid organ transplant recipients does not totally exclude CMV disease, because compartmentalized or localized CMV diseases have very low or transient periods of viraemia.^18^, ^19^, ^20^ It is uncommon for encephalitis to present with a negative PCR test for blood CMV and in the absence of CNS disease symptoms.^15^, ^21^ The absence of symptoms of CNS infection in this case is probably because of the lack of lymphocytes and the state of profound anergy induced in the recipient. The virus cannot *per se* cause visible lesions without the host inflammatory response.^21^

It is plausible that the patient had an asymptomatic infection, clinically undetectable during the post‐transplant phase of profound lymphopenia. With the subsequent reconstitution of her immune system, however, the immune response to the pathogen produced symptoms that unmasked the infection. This is a sequence of events well described in HIV‐positive patients.^22^, ^23^ The most common viral cause of IRIS is polyomavirus JC, but CMV can be a trigger.^11^

The patient’s symptoms, the high CSF viral load and the elevated levels of IL‐6 in the CSF could all be consistent with enhanced permeability of the blood–brain barrier (BBB) because of inflammation, which would allow certain molecules to cross it in small amounts; this has been demonstrated in animal models.^24^ BBB inflammation may also explain the initial rapid regression of symptoms at the start of antiviral therapy with ganciclovir and foscarnet, although they are known to have a poor BBB penetration.^25^, ^26^ The subsequent restoration of BBB impermeability may explain why the CSF viral load subsequently remained almost unchanged despite the double antiviral therapy.

We suggest that high dosage CMV‐hyperimmune globulin succeeded in achieving CMV clearance because of immunoglobulins’ ability for BBB penetration. Murine models show that specific antibodies may cross the BBB in the context of a viral CNS infection and that specific anti‐CMV antibodies may have a protective role in reducing viral lesions in brain tissue.^24^ Furthermore, aseptic meningitis, an adverse effect of immunoglobulin treatment, may be because of a hypersensitivity reaction to the direct entry of a small quantity of immunoglobulin into the CNS compartment.^27^, ^28^

Blood immunoglobulins have to cross two different barriers to reach the CSF: the BBB and the blood–CSF barrier. A study reported that the concentration of injected humanized IgGs in rats decreased more slowly in the blood than in the CSF, showing that IgGs, once administered into the intra‐cerebroventricular (ICV) space, are cleared by bulk flow, disappearing from CSF within 24 h with a half‐life of 47.0 ± 6.49 min and a clearance of 29.0 ± 15.2 mL day^−1^ kg^−1^, comparable to the rate of inulin.^29^

To prevent a reactivation of latent CMV infection, patients are treated with Cytotect CP, an immunoglobulin preparation from donor plasma with a high antibody titre against CMV. It is well known that only a small fraction of injected immunoglobulins can cross the BBB, with a study reporting that 0.009 ± 0.001% and 0.0017 ± 0.0005% of systemically administered IgGs reached the cortex and hippocampus of animals after a single injection.^30^

Although encephalitis is associated with an increase of BBB permeability, the diffusion of molecules follows the concentration gradient, and for that reason, we should not detect a concentration of IgGs in CSF greater than that found in plasma.

These data strongly support the view that when antibody levels are significantly higher in CSF than in plasma, it is not because of a decreased clearance from the CSF, but more likely because of an intrathecal production of new antibodies. While it is not surprising to find a very high IgG antibody titre (230 U mL^–1^) in a patient suffering from a CMV encephalitis, it is interesting to note that patients who undergo prophylaxis with Cytotect CP also have a greater titre of anti‐CMV‐IgG in CSF than in plasma. After intravenous administration of anti‐CMV‐IgGs, the small amount of immunoglobulins that crosses the BBB is probably able to trigger the local production of anti‐CMV‐IgG. This offers confirmation of a protective role for intravenous immunoglobulins.

## Conflict of interest

The authors have declared that no conflict of interest exists.

## Funding

No specific funding was received.

## Author Contributions

**Natalia Maximova:** Conceptualization; Data curation; Writing‐original draft. **Annalisa Marcuzzi:** Formal analysis; Investigation; Methodology. **Irene Del Rizzo:** Validation; Visualization; Writing‐original draft. **Davide Zanon:** Resources; Supervision. **Alessandra Maestro:** Data curation; Investigation. **Egidio Barbi:** Project administration; Writing‐review & editing. **Roberto Sala:** Supervision; Writing‐review & editing.
